# Experimental and CFD Investigation of Nanofluid-Based Cooling Performance in an Automotive Radiator Under Real Operating Conditions

**DOI:** 10.3390/nano16140844

**Published:** 2026-07-09

**Authors:** Beytullah Erdoğan, Güneyhan Taşkaya

**Affiliations:** Department of Mechanical Engineering, Engineering Faculty, Zonguldak Bülent Ecevit University, Zonguldak 67100, Turkey; guneyhantaskaya@gmail.com

**Keywords:** nanofluid, CFD, cooling system, automobile radiator, hybrid nanofluid

## Abstract

In this study, the cooling performances of various nanofluids were compared under the operating conditions of a real automobile radiator, based on an internal combustion engine vehicle cooling system whose experiments had been previously completed. In the analyses, the radiator inlet fluid temperature was fixed at 70 °C, air inlet velocities were set to 6, 8, and 10 m/s, and fluid flow rates were taken as 17, 19, and 21 L/min. Under these conditions, the cooling capacities were evaluated for three different working fluids whose thermophysical properties were experimentally determined: 100% pure water, water-based 0.3% ZnO nanofluid, and water-based 0.3% ZnO + CuO hybrid nanofluid. Within the scope of this study, a Computational Fluid Dynamics (CFD) model was developed based on the aforementioned experimental parameters and validated with a maximum deviation of 6%. Using the validated model, additional CFD analyses were performed for water-based 0.3% Al_2_O_3_ and TiO_2_ nanofluids, whose thermophysical properties were also experimentally determined, and their cooling performances were assessed. Based on the experimental and numerical results obtained, the highest cooling capacity was determined to be 20.8 kW in the 0.3% TiO_2_ nanofluid, representing a 69.1% increase in cooling capacity compared to pure water. These findings clearly demonstrate that the use of nanofluids significantly enhances heat transfer performance in automotive cooling systems.

## 1. Introduction

In the modern automotive industry, the increase in power density of internal combustion engines, the trend toward more compact vehicle designs, and stringent Euro emission regulations have made thermal management a critical challenge [1]. In internal combustion engines, fuel efficiency, energy efficiency, and emission reduction are directly dependent on maintaining the engine temperature within an optimal range. At this point, the radiator serves as the most essential component of the cooling system by dissipating the excess heat generated by the engine. However, conventional coolants such as water or ethylene glycol have relatively low thermal conductivity, limiting their ability to transfer heat efficiently. Studies conducted in recent years have shown that, thanks to their enhanced thermophysical properties, nanofluids can significantly contribute to the development of more compact and efficient cooling systems by increasing heat transfer in automotive radiators [2,3]. This necessitates the use of larger radiators while also leading to reduced system efficiency [4,5].

Nanofluids are advanced working fluids obtained by dispersing nanoscale particles into conventional base fluids such as water or ethylene glycol. Examples include Al_2_O_3_, CuO, ZnO, TiO_2_, graphene, and various nanoparticle mixtures [6,7,8]. Compared to pure water and ethylene glycol, nanofluids exhibit enhanced thermal conductivity, enabling more efficient heat transfer [9]. Studies that numerically investigate the behavior of hybrid and triple-hybrid nanofluids in various flow and heat transfer problems have shown that the thermophysical properties of nanofluids can significantly improve heat transfer performance [10,11]. These developments further enhance the potential for using nanofluids in engineering applications such as automotive cooling systems. Recent experimental studies have demonstrated that radiators utilizing nanofluids can achieve heat transfer enhancements ranging from 10% to 50% compared with those using pure water or ethylene glycol [12,13,14]. In experimental investigations, internal and external flows in automotive cooling systems are generally examined separately. Internal flow studies focus on parameters such as nanoparticle type, concentration, inlet fluid temperature, and flow rate. External flow analyses, on the other hand, investigate the effects of radiator geometry, fin configuration, air velocity, and airflow guiding structures positioned in front of the radiator [15,16,17,18]. Although increasing nanoparticle concentration generally improves heat transfer, excessive concentrations may lead to agglomeration, sedimentation, and increased pressure drop. Furthermore, optimization of operating conditions such as flow rate and temperature difference is crucial for maximizing the ratio of heat transfer to pumping power [19].

In addition to energy efficiency, exergy analysis has recently become an important area of study in cooling systems. Exergy analysis evaluates irreversibilities and entropy generation occurring during energy conversion processes, thereby determining the thermodynamic perfection of a system [20]. By assessing the useful work potential of energy, this method provides a comprehensive evaluation of system performance from a second-law efficiency perspective. Experimental energy–exergy analyses have shown that nanofluids not only enhance heat transfer but also improve the overall thermodynamic efficiency of the system [21]. Relying solely on experimental methods may be insufficient to fully capture the complex flow and temperature distributions in intricate radiator geometries, as such approaches require considerable time, high cost, and specialized experimental setups. Moreover, limitations related to sensor placement, cost, time, and repeatability often make it impractical to experimentally test all parameter combinations. Therefore, in recent years, Computational Fluid Dynamics (CFD) has emerged as a powerful complementary tool to experimental studies. CFD enables detailed analysis of flow fields, temperature distributions, pressure drops, and heat transfer mechanisms by numerically solving the governing equations of continuity, momentum, and energy [6,22,23].

Through CFD analyses, the effects of parameters such as radiator geometry, fin structure, channel design, thermophysical properties of nanofluids (including thermal conductivity, dynamic viscosity, specific heat, and density), and flow direction on system performance can be investigated without the need for prototype manufacturing. Furthermore, CFD can provide highly realistic results by accounting for temperature-dependent thermophysical properties, multiphase flow effects, and the non-Newtonian behavior of nanofluids. These models reveal local temperature gradients, turbulence effects, and pressure losses in detail, and when combined with experimental observations, they serve as powerful optimization tools [24,25]. The increase in convective heat transfer observed in nanofluids is associated with various physical mechanisms, such as the micro-mixing mechanism caused by Brownian motion, particle migration, microconvection effects, boundary layer thinning, and turbulence interactions. In particular, it has been reported that Brownian motion can increase local energy transport within the fluid, while the migration of nanoparticles in the flow direction can alter the thermal boundary layer structure by affecting temperature gradients. Furthermore, some studies indicate that the addition of nanoparticles can provide additional improvements in the convective heat transfer coefficient by altering the flow behavior near the wall [26,27].

This study investigates the thermal performance of an automotive radiator operating under realistic engine cooling conditions (70 °C coolant inlet temperature, 17–21 L/min coolant flow rates, and 6–10 m/s air velocities) using pure water, mono nanofluids (Al_2_O_3_ and TiO_2_), and hybrid nanofluids. Although there are numerous experimental and numerical studies in the literature on the use of nanofluids in automotive cooling systems, comprehensive comparative studies conducted under operating parameters that represent actual engine-operating conditions using a commercial automotive radiator are limited. Furthermore, a significant portion of existing studies typically focuses on a single type of nanofluid, simplified radiator geometries, or idealized operating conditions; no study has been found that simultaneously employs all of the following in a single study: real radiator geometry, CFD validation of experiments conducted under real operating conditions, and the use of all types of hybrid nanofluids containing Al_2_O_3_, TiO_2_, and ZnO. From this perspective, the article offers a comprehensive, reproducible, and highly economical method for the exploration of different types of nanofluids. In addition, the study presents a comparative investigation of mono and hybrid nanofluids in an automotive cooling application, offering further insight into their effects on cooling load, thermal performance, and exergy behavior. The proposed experimental–numerical methodology contributes to the understanding of nanofluid-based radiator systems and provides a useful basis for future optimization and design studies in automotive thermal management.

## 2. Materials and Methods

### 2.1. Selection, Preparation, and Measurement of Thermophysical Properties of Nanofluids

In this study, both experimental and numerical analyses were carried out on a radiator used in an internal combustion engine cooling system. In the first stage, pure water, water-based ZnO, and water-based ZnO + CuO hybrid nanofluids, which had been previously investigated experimentally in the cooling system, were utilized. Based on the obtained experimental data, the system was validated using the Computational Fluid Dynamics (CFD) approach. Following model validation, water-based Al_2_O_3_ and TiO_2_ nanofluids were prepared and incorporated into the analyses. Within the scope of this study, Al_2_O_3_, TiO_2_, ZnO, and ZnO + CuO hybrid nanoparticles were employed. The primary reason for selecting these nanoparticles is their complementary thermophysical properties. ZnO and CuO nanoparticles have the potential to enhance heat transfer performance due to their high thermal conductivity, whereas Al_2_O_3_ nanoparticles stand out as a reliable reference material owing to their low cost, good dispersion characteristics, and widespread use in the literature. On the other hand, TiO_2_ nanoparticles contribute to system stability due to their chemical stability and low tendency toward agglomeration. Detailed physical and thermal properties of ZnO, CuO, Al_2_O_3_, and TiO_2_ nanoparticles, as provided by the manufacturer (Merck KGaA, Darmstadt, Germany), are presented in Table 1 [28,29].

In nanofluid preparation, one-step and two-step methods are generally employed. In the one-step method, nanoparticle synthesis and dispersion occur simultaneously, whereas in the two-step method, pre-synthesized nanoparticles are dispersed into the base fluid. In this study, the two-step method was preferred due to its ease of application and cost-effectiveness. The nanoparticles were procured from commercial suppliers and added to deionized water at a total volumetric concentration of 0.3%. The nanoparticles were gradually introduced into the base fluid under continuous stirring to reduce particle agglomeration before the ultrasonication process. To enhance stability, Sodium Dodecyl Sulfate (SDS), one of the most commonly used surfactants in the literature, was added at a ratio of 50% of the total nanoparticle mass. For homogeneous dispersion, ultrasonic treatment was applied for 30 min using a probe-type ultrasonic homogenizer (Optic Ivymen System/CY-500; 500 W, 20 kHz, Barcelona, Spain), resulting in stable nanofluids. The same preparation procedure, including nanoparticle concentration, surfactant ratio, and ultrasonication conditions, was adopted as in our previous optimization study [29] to ensure reproducible dispersion quality. The thermal conductivity, dynamic viscosity, and density values of water-based Al_2_O_3_, TiO_2_, ZnO, and CuO + ZnO nanofluids prepared at a volumetric concentration of 0.3% were experimentally measured at temperatures of 293, 303, 313, and 323 K, and are presented in Table 2. A thermostatic water bath (JSR JSIB-11T; 5–99 °C, 12 L·min^−1^) was used to maintain stable temperature conditions. Thermal conductivity measurements were performed using a KD2 Decagon Pro (0.02–2.00 W/m·K, ±5–±10% accuracy, −50–150 °C, Meter Group, Pullman, WA, USA). Dynamic viscosity was measured using a FUNGILAB Smart L viscometer (2000–21,333 mPa·s, 16 mL sample, FUNGILAB S.A., Sant Feliu de Llobregat, Barcelona, Spain), while density was determined using an Anton Paar DMA 35 densitometer (0–2 g/cm^3^, 50 mL sample, Anton Paar GmbH, Graz, Austria). Each measurement was repeated at least three times for each temperature, and the average values were reported. The specific heat capacity was calculated using the experimentally obtained density data and Equation (1), where *ρ_np_*, *c_np_*, *ϕ*, *ρ_bf_*, and *C_bf_* represent the nanoparticle density, specific heat, volumetric concentration, base fluid density, and base fluid specific heat, respectively. The measured values of density, viscosity, and thermal conductivity were compared with relevant studies in the literature [30]. The results obtained were found to be consistent with the literature. It is believed that the minor differences observed may stem from variations in nanoparticle size, dispersion method, and temperature conditions. Measurements of thermal conductivity, dynamic viscosity, density, zeta potential, and particle size were conducted to evaluate the effect of each surfactant (SDS) used. Subsequently, all results were compared with those of the prepared pure base fluid. In a previous stability study conducted using the same nanoparticle concentration, surfactant ratio, and ultrasonication conditions, the nanofluid maintained its stable structure for up to 7 days [29,31]. SDS can indirectly influence boundary layer development and convective heat transfer behavior by altering interfacial properties such as surface tension and wettability, in addition to enhancing nanoparticle stability. Therefore, it is assessed that the observed improvement in heat transfer may stem not only from the nanoparticle contribution but also from the surfactant effect. The ultrasonic mixing time and detailed stability characterizations, such as Zeta potential or TSI, were obtained from a previous literature study [32]. The objective is to ensure the homogeneous distribution of nanoparticles and minimize agglomeration. The prepared nanofluids were used within the stability period reported in our previous study [32], in which the same preparation protocol was employed. However, no visible precipitation, phase separation, or noticeable sedimentation was observed throughout the experimental period. In addition, the good repeatability of the thermophysical property measurements confirmed that the nanofluids remained sufficiently stable during all experiments.

(1) $$\rho_{nf}c_{nf}=\rho_{np}c_{np}\phi +\rho_{bf}c_{bf}\left(1-\phi \right)$$

### 2.2. Governing Equations

To determine the flow regime, the Reynolds number (Re), a dimensionless parameter defined for flows within pipes and channels, was calculated using the coolant flow rate in the system and the average air velocity acting on the radiator, as suggested by Çengel et al. [33]. The Reynolds number is expressed as in Equation (2):

(2) Re = u Dhv = Inertial ForcesViscous Forces

Here, *u* represents the fluid velocity (m/s), *D_h_* represents the hydraulic diameter of the channel (m), and *v* represents the kinematic viscosity of the fluid. The dimensionless Prandtl number (Pr), which describes the relative development of hydrodynamic and thermal boundary layers, is defined as the ratio of momentum diffusivity to thermal diffusivity and is given in Equation (3). In this expression, *α* represents the thermal diffusivity:

(3) $$\mathrm{Pr}\;=\;\frac{\upsilon }{\alpha }=\;\frac{Momentum\;Diffusivity}{Thermal\;Diffusivity}$$

Here, *α* represents the thermal diffusion coefficient (m^2^/s). The thermal diffusivity is defined as *α* = *k*/*ρc_p_*.

When *Pr* > 1, the hydrodynamic boundary layer develops faster than the thermal boundary layer, whereas *Pr* = 1 indicates that both boundary layers develop simultaneously. In flows within pipes and channels, heat transfer occurs primarily through convection, and the Nusselt number (*Nu*) is a key parameter used to determine the convective heat transfer coefficient. For forced convection in internal flows, the Nusselt number is related to the Reynolds and Prandtl numbers as well as parameters depending on the channel geometry. In other words, the Nusselt number represents the ratio of convective to conductive heat transfer and is given in Equation (4). The hydraulic diameter used in the Nusselt number expression is calculated as presented in Equation (5).

(4) $$\mathrm{Nu}=\frac{hD_{h}}{k}=\frac{Convective\;Heat\;Transfer}{Conductive\;Heat\;Transfer}$$

(5) $$D_{h}=\frac{4\times Area}{Perimeter}$$

Here, *h* represents the heat transfer coefficient (W/m^2^K). According to Newton’s law of cooling, as described by Çengel [34], the heat transfer for the coolant is calculated using Equations (6) and (7).

Q˙_sa_ = *m*˙_sa_ × *c*_*p*,sa_ × (*T*_sa,in_ − *T*_sa,out_) (6)

m˙_sa_ = ρ_sa_ × V_sa_ × A (7)

For the airflow, the required calculations are performed using Equations (8) and (9).

m˙_h_ = ρ_h_ × V_h_ × A (8)

Q˙_h_ = *m*˙_h_ × *c*_*p*,h_ × (*T*_h,in_ − *T*_h,out_) (9)

In heat exchangers, the overall heat transfer coefficient is calculated by considering both convective and conductive thermal resistances, as expressed in Equation (10).

(10) $$\frac{1}{UA}=\frac{1}{U_{h}A_{h}}=\frac{1}{U_{c\;}A_{c}}=\frac{1}{{\left(hA\right)}_{h}}+R_{w}+\frac{1}{{\left(hA\right)}_{c}}$$

In heat transfer analysis, when fins are attached to both sides of the surfaces, the formulation is extended accordingly and expressed as given in Equation (11).

(11) $$\frac{1}{UA}=\frac{1}{{\eta_{o}(hA)}_{hot}}+R_{w}+\frac{1}{{\eta_{o}(hA)}_{cold}}$$

In the equation, the term $\eta_{o}$ represents the overall surface efficiency (surface effectiveness), which is determined based on the efficiencies of all fins ($\eta_{f}$). The overall surface efficiency $\eta_{o}$ is calculated using the heat transfer relation given in Equation (12).

(12) $$Q˙=\eta_{o}hA(T_{b}-T_{\infty })$$

The overall heat transfer coefficient is determined based on the heat transfer coefficients of the fluids, fouling factors, and geometrical properties [35]. In this context, *T_b_* denotes the base surface temperature, *A* represents the surface area, and R_w_ refers to the wall thermal conduction resistance for clean surfaces. Fouling in heat exchangers increases thermal resistance, thereby reducing performance and introducing an additional thermal resistance term known as the fouling factor (R_f_). These factors depend on operating temperature, fluid flow rate, and the service life of the heat exchanger. The overall heat transfer coefficient is expressed by Equation (13).

(13) $$\frac{1}{UA}=\frac{1}{U_{h}A_{h}}=\frac{1}{U_{c\;}A_{c}}=\frac{1}{{\eta_{o}(hA)}_{h}}+\frac{R_{f,h}}{{\eta_{o}(hA)}_{h}}+R_{w}+\frac{1}{{\eta_{o}(hA)}_{c}}+\frac{R_{f,c}}{{\eta_{o}(hA)}_{c}}$$

In Equation (12), the subscript *h* denotes the hot fluid, while the subscript *c* refers to the cold fluid. The overall surface efficiency can be expressed as a function of fin efficiency and is calculated using Equation (14).

(14) $$\eta_{o}=1-\frac{A_{f}}{A}\left(1-\eta_{f}\right)$$

In Equation (14), *A_f_* represents the total fin surface area, while *η_f_* denotes the efficiency of a single fin. For a fin with an insulated tip, the fin efficiency is given by Equation (15). The heat transfer coefficient of fins varies depending on the material and geometric arrangement; however, in practice, it is commonly assumed to be equal to the heat transfer coefficient of the unfinned surface [32].

(15) $$\eta_{f}=\frac{tanh(mL)}{mL}$$

A Computational Fluid Dynamics (CFD) model was employed within the radiator control volume. A pressure-based solver was selected to simulate the flow inside the radiator. The motion of the fluid within the control volume is described by the continuity equation. The conservation of mass (continuity) equation for the system is expressed in Equation (16).

(16) $$\frac{\partial \rho }{\partial t}+\frac{\partial (\rho v_{i})}{\partial x_{i}}=0$$

Here, *ρ* denotes density, *t* represents time, and *v_i_* refers to the velocity components. The momentum and energy equations are given in Equations (17) and (18), respectively. In these equations, *v_i_*, *T*, *P*, *μ*, *C_p_*, and *k* represent the velocity, temperature, pressure, dynamic viscosity, specific heat capacity, and thermal conductivity, respectively.

(17) $$\frac{\partial (\rho v_{i}v_{j})}{\partial x_{j}}=-\frac{\partial P}{\partial x_{i}}+\frac{\partial }{\partial x_{j}}\left[\mu \left(\frac{\partial v_{i}}{\partial x_{j}}+\frac{\partial v_{j}}{\partial x_{i}}\right)-\frac{2}{3}\mu \frac{\partial v_{k}}{\partial x_{k}}\delta_{ij}\right]$$

(18) $$\frac{\partial (\rho e)}{\partial t}+\frac{\partial }{\partial x_{j}}\left(\rho v_{j}C_{p}T-k\frac{\partial T}{\partial x_{j}}\right)=u_{j}\frac{\partial P}{\partial x_{j}}+\left[\mu \left(\frac{\partial v_{i}}{\partial x_{j}}+\frac{\partial v_{j}}{\partial x_{i}}\right)-\frac{2}{3}\mu \frac{\partial v_{k}}{\partial x_{k}}\delta_{ij}\right]$$

### 2.3. Experimental Setup

**Figure 1:**
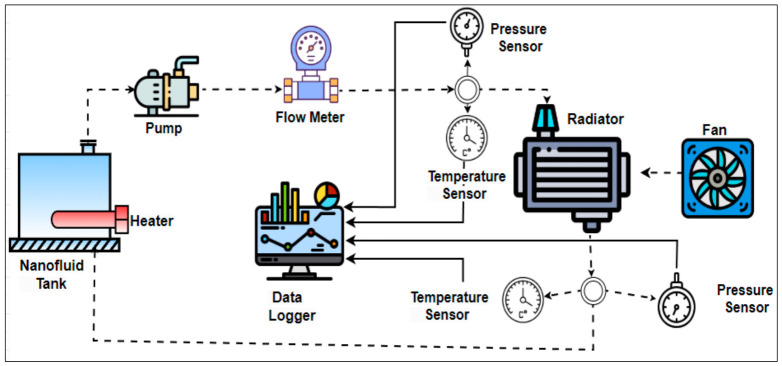
Experimental setup.

**Figure 2:**
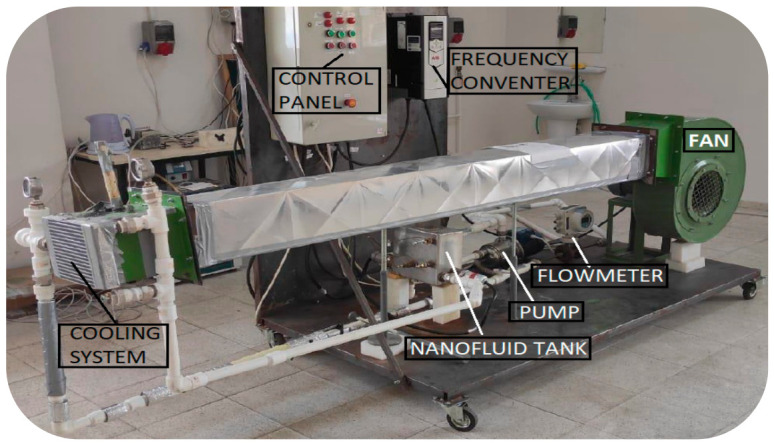
Experimental Setup [28].

The experimental system used in this study was designed to evaluate the cooling performance of automotive radiators with different types and geometries under real operating conditions. Typical parametric conditions of internal combustion engine vehicles were considered in the experimental setup. In this context, the coolant inlet temperature was kept constant at 70 °C, while the fluid flow rates were selected as 17, 19, and 21 L/min, and the external air velocities were set to 6, 8, and 10 m/s. Radiator performance tests were conducted within these operating ranges. In addition, different types of nanofluids, along with the base fluid, were used to comparatively investigate variations in cooling performance. The experimental setup shown in Figure 1 and Figure 2 consists of a nanofluid tank, a heater used to maintain the inlet fluid temperature at 70 °C, and a pump to ensure fluid circulation. The system is equipped with a flow meter to control different flow rates (17, 19, and 21 L/min), as well as pressure and temperature sensors placed at the inlet and outlet of the cooling system. In front of the radiator, a fan system is installed to simulate external airflow at velocities of 6, 8, and 10 m/s, along with a frequency inverter to control the fan speed. The electric heater in the nanofluid tank assists in achieving the desired inlet temperature conditions.

The pump continuously circulates the nanofluid through the radiator, while the flow rate and pressure values are monitored using a flow meter and pressure sensors. Temperature sensors located at the inlet and outlet of the radiator record the temperature variations in the fluid during the heat transfer process. All measured data are collected via a computer-based data acquisition (data logger) system, enabling energy and exergy analyses of the system. The fan unit simulates the external airflow, thereby enhancing the cooling performance by promoting air movement through the radiator. Overall, this experimental setup enables a comprehensive investigation of radiator performance under various nanofluid types with different concentration ratios, fluid and air velocity conditions, and inlet temperature levels.

### 2.4. Computational Setup

**Figure 3:**
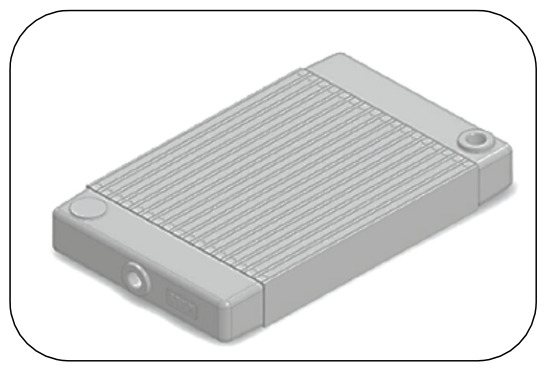
3D model geometry of the automotive radiator used in this study [28].

**Figure 4:**
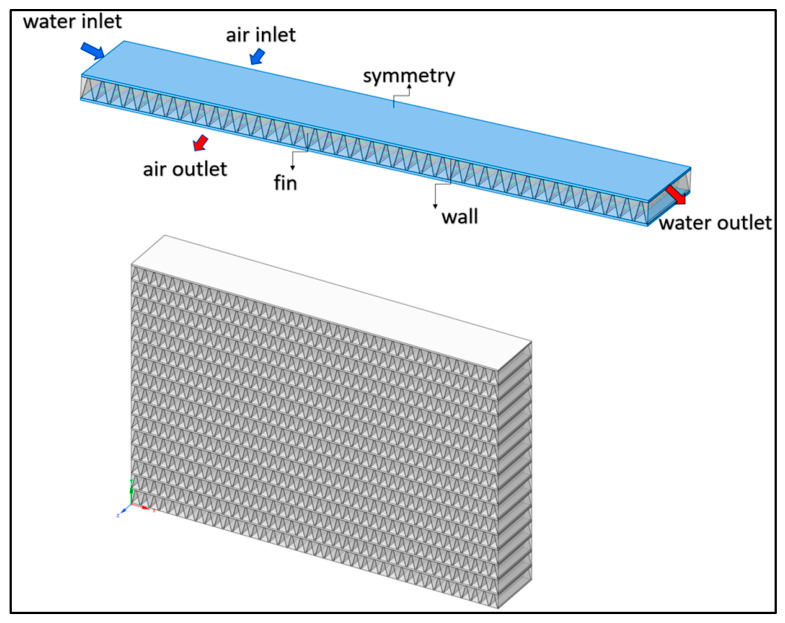
Computational geometry and boundary conditions of the analysis.

In this study, a KALE/ABB automotive radiator, whose technical specifications and geometrical dimensions are provided in Table 3, was used. The solid model of the radiator, shown in Figure 3, consists of 16 flow channels. The flow domain and boundary conditions of the radiator geometry used in the numerical analysis are presented in Figure 4. The three-dimensional model created using ANSYS (2024 R2 ANSYS Inc., Canonsburg, PA, USA) Fluent consists of two distinct flow zones: water and air. In order to ensure a uniform and symmetric distribution of the airflow, the air region of the radiator was modeled as a finned structure. The mesh structure was generated using the finite volume approach in ANSYS Fluent Meshing, and symmetric modeling was adopted to reduce computational cost and improve calculation efficiency.

The numerical model was developed based on the actual dimensions of the radiator. However, due to the geometric complexity and computational difficulties, a single channel was modeled using a symmetry approach. To reduce computational costs, the single-channel symmetry approach—commonly used in radiator CFD studies in the literature—was adopted and is presented in Figure 4 [36]. However, in real radiator systems, irregularities in the airflow, manifold effects, and differences in flow distribution can influence full-scale behavior. Therefore, the current model provides an idealized approach. The model consists of inlet, outlet, wall, and symmetry boundary conditions. In regions close to the walls, a five-layer boundary layer mesh was employed to capture velocity gradients resulting from flow deceleration. The numerical model was constructed to evaluate the cooling load of the radiator under the specified boundary conditions, taking into account both liquid flow and air velocity effects. The air inlet represents the airflow passing through the finned structure, while the water inlet defines the boundary conditions for the coolant flowing through the channels. In addition, fin geometries were incorporated to enhance heat transfer between air and water. The effect of adding three different types of nanoparticles into the coolant flowing through the channels was also numerically modeled in terms of heat transfer performance. The thermophysical properties of fluids have been incorporated into the model as functions of temperature.

**Figure 5:**
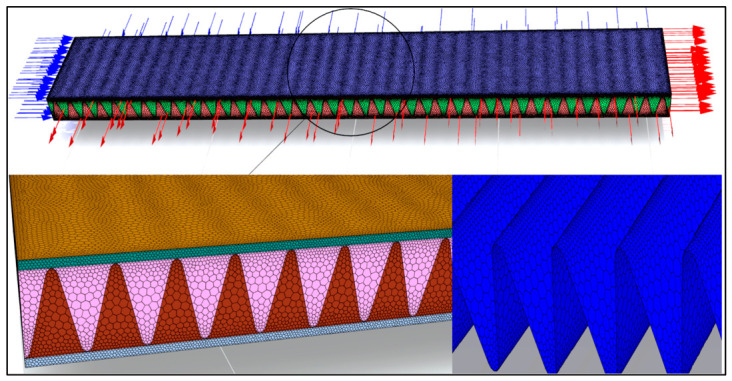
Mesh structure of the computational model.

The number of mesh elements in the three-dimensional computational model is one of the most critical factors affecting CFD accuracy. While increasing the mesh density improves solution accuracy, it also increases computational time; conversely, insufficient mesh resolution reduces the reliability of the results. Therefore, an optimal mesh size is essential. For the symmetric model, approximately 1.2 million (1.2 M) mesh elements were used. At this mesh density, the average deviation between numerical and experimental results was found to be approximately 3%, indicating that the mesh resolution was sufficient. Figure 5 shows the mesh structure generated for the numerical model.

**Figure 6:**
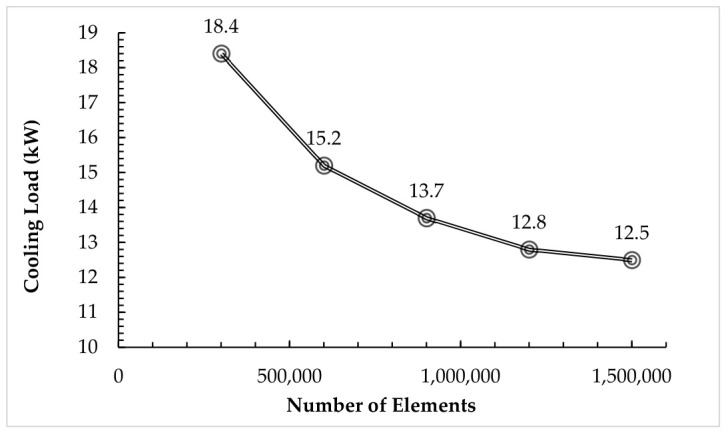
Study on the independence of the mixture obtained from Experiment 1 with water.

The study on mesh independence conducted using the First Water Experiment is shown in Figure 6. The standard wall function was used as the wall model, with a convergence criterion of 10^−3^ for continuity and momentum, 10^−7^ for the energy equation, a second-order upwind discretization scheme, and the SIMPLE/Coupled algorithm for pressure-velocity coupling. Boundary layer development and turbulence effects in the gas-side flow were taken into account in the modeling. The realizable k-ε turbulence model was used in the study. Flow behavior near the wall was solved using wall functions, and the boundary layer resolution was ensured in accordance with the y+ criteria.

## 3. Results and Discussion

This section presents the cooling performance results obtained from the nine experimental cases conducted under different working conditions, including fluid type (pure water, ZnO + water, and ZnO + CuO + water), flow rate (17, 19, and 21 L/min), and air velocity (6, 8, and 10 m/s), as summarized in Table 4. The evaluated results indicate that, for all working fluids, an increase in both volumetric flow rate and air velocity leads to a direct enhancement in the cooling load (Q_cool_). The performance of pure water varies between 9.8 kW and 12.9 kW, whereas the use of nanofluids significantly improves these values. In particular, the highest cooling performance of 19.6 kW was achieved in Test 9, where the ZnO + CuO + water hybrid nanofluid was used under conditions of 21 L/min flow rate and 10 m/s air velocity. This result demonstrates that the thermal conductivity advantage of nanofluids, when combined with higher flow velocities, can increase the cooling capacity by more than 50% compared with pure water, thereby significantly enhancing the efficiency of thermal systems.

**Figure 7:**
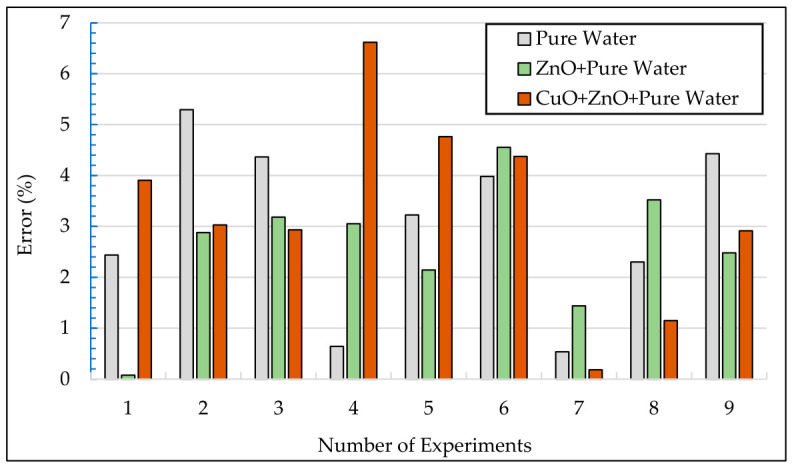
Error rates between experimental and CFD results.

Figure 7 presents the validation results of the CFD simulations against the experimental data for the cooling load of pure water, ZnO + water, and hybrid ZnO + CuO nanofluids. The results obtained under nine different operating conditions demonstrate that the error rates remain below the 7% threshold, indicating that the developed model has a high level of reliability from an academic perspective. A detailed examination of the results shows that the lowest error rate, approximately 0.1%, occurs under Test 1 conditions (70 °C, 17 L/min, 6 m/s) for the ZnO + water mixture. In contrast, the highest deviation is recorded in Test 4 (70 °C, 19 L/min, 6 m/s) for the ZnO + CuO + water hybrid nanofluid, with an error of approximately 6.6%. It is also observed that the error rates for pure water vary depending on the operating conditions; however, under higher flow rate and air velocity conditions (Tests 6 and 9), the error values for all working fluids stabilize within the range of approximately 2.5% to 5.5%.

Based on these findings, it can be inferred that the addition of nanoparticles increases the complexity of the thermophysical properties of the working fluid, introducing a certain level of uncertainty in the modeling process. Nevertheless, the overall error distribution remaining within acceptable engineering limits confirms the accuracy of the simulations and the validity of the model. In this study, the cooling loads of the working fluids were evaluated under conditions of 21 L/min flow rate, 70 °C inlet temperature, and 10 m/s air velocity, and the results are presented in Table 5.

**Figure 8:**
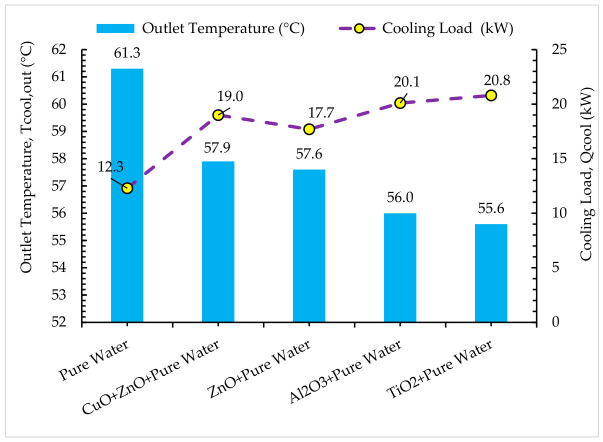
Comparison of cooling load and outlet temperature of nanofluids.

Following the validation studies, CFD analyses were performed for water-based Al_2_O_3_ and TiO_2_ nanofluids whose thermophysical properties were experimentally determined. The obtained results, presented in Table 5 and Figure 8, clearly illustrate the effects of different working fluids on cooling load and outlet temperature. Compared to pure water, the use of nanofluids was found to enhance heat transfer performance. In particular, the cooling load reached approximately 20.1 kW and 20.8 kW for the Al_2_O_3_ + PW and TiO_2_ + PW mixtures, respectively. In contrast, pure water exhibited a lower cooling load (12.3 kW) and a higher outlet temperature (61.3 °C). Among the nanofluids, the outlet temperatures were observed to vary in the range of 55.6–56.0 °C. These findings indicate that the addition of nanoparticles improves the thermal performance of the working fluid, resulting in more effective cooling and enhanced system efficiency.

**Figure 9:**
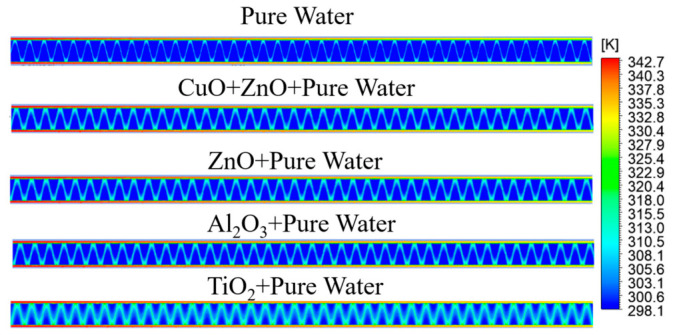
Temperature contours of nanofluids.

The temperature variations in the nanofluids prepared with different water-based nanoparticles within the radiator channels and fins are presented in Figure 9, where the temperature contours exhibit a clear distribution ranging from 298.1 K to 342.7 K. Along the radiator channels, the fluid cools progressively from the high inlet temperature toward the outlet due to heat transfer, with nanoparticles possessing high thermal conductivity playing an effective role in this process. Compared with pure water, it is observed that CuO + ZnO-, ZnO-, Al_2_O_3_-, and TiO_2_-based nanofluids generate a more uniform and widespread heat distribution over the fin structures. In particular, in channels where hybrid and metal-oxide-based fluids are used, the more effective dissipation of thermal energy to the fin surfaces demonstrates the enhancement of heat transfer performance by nanofluids. As a result, the color variations over the fin surfaces indicate that nanofluids exhibit superior cooling performance compared to pure water in terms of heat removal from the radiator surface and optimization of cooling efficiency.

## 4. Uncertainty Analysis

Uncertainty analyses are necessary to interpret data obtained from experimental studies with high accuracy and to enhance the reliability of the study. Despite the high precision of measuring instruments, deviations may occur in measurements due to various sources of error, and this can make it difficult to determine the true values with complete accuracy. In this study, the uncertainties in the input parameters and the error rates of the measuring instruments were determined, and the error margins of the devices measuring fluid properties were also included in the calculations. Uncertainties in the calculated heat transfer rate, used to evaluate the system’s cooling performance, were also analyzed. The uncertainty analysis in this study was addressed under two main headings: (i) uncertainties associated with directly measured variables such as temperature and pressure, and (ii) uncertainties associated with derived variables calculated using the measured data. In this context, experimental uncertainties were estimated using the method proposed by Kline and McClintock [37]. As a result, the maximum uncertainties for heat transfer and pressure drop were determined to be 26.25% and 12.73%, respectively. These values are presented in Table 6 and Table 7.

The calculated maximum uncertainties for thermal conductivity, dynamic viscosity, density, and specific heat are 6.13%, 7.26%, 0.19%, and 2.50%, respectively. To understand the influence of these uncertainties on the numerical results, sensitivity analyses were conducted by varying the thermophysical properties within their maximum uncertainty margins.

## 5. Conclusions

In this study, the effects of different nanofluids and operating parameters on the cooling performance of a radiator system were investigated using both experimental and numerical (CFD) methods. The obtained results demonstrate that increasing volumetric flow rate and air velocity directly enhances the cooling load for all working fluids.

- In the experimental investigations, the cooling performance of pure water remained in the range of 9.8–12.9 kW, whereas the use of a ZnO + CuO + water hybrid nanofluid under conditions of 21 L/min flow rate and 10 m/s air velocity resulted in a maximum cooling load of 19.6 kW, corresponding to an improvement of approximately 50%.
- The validation of the developed CFD model against experimental data showed that the error rates remained below 7%, confirming the academic reliability of the model.
- Additional CFD analyses revealed that TiO_2_ and Al_2_O_3_ nanofluids increased the cooling load to 20.8 kW and 20.1 kW, respectively, while significantly reducing the outlet temperature compared to pure water. Temperature contour results further confirmed that nanofluids provide a more uniform heat distribution over the radiator fins, thereby enhancing heat transfer efficiency.

The results of this study demonstrate that nanofluids have the potential to improve engine cooling efficiency by enhancing heat transfer performance in automotive radiators and enabling more compact radiator designs. However, for nanofluids to be widely used on a commercial scale, important factors such as the increased pumping power required due to higher viscosity, corrosion and wear effects, and production costs must also be evaluated. In conclusion, the addition of nanoparticles improves thermophysical properties, increases system efficiency, and offers a strong alternative for high-performance cooling systems. This study offers the literature a comprehensive, reproducible, and cost-effective method for discovering new types of nanofluids with high thermophysical properties, which are essential for the design of high-efficiency automotive radiators. Future studies should focus on the use of ternary (three-component) nanofluids and investigate the thermal effects of different nanoparticle combinations to further enhance internal flow performance. In addition, for external flow improvement, geometric modifications of the radiator front panel are recommended to maximize airflow dynamics and overall cooling efficiency.

## Figures and Tables

**Table 1:** Properties of Nanoparticles [28,29].

Nanoparticle	Purity	Particle Dia. (nm)	Geometry	Density (kg/m^3^)	Spec Heat (J/kgK)	Thermal Cond. (W/mK)
Al_2_O_3_	%99.8	13–20 nm	Spherical	3890	778	46
TiO_2_	%99.5	10–25 nm	Spherical	3900	710	10
ZnO	%99.5	40 nm	Spherical	5500	544	19
CuO	%99.8	30–50 nm	Spherical	6500	540	33

**Table 2:** Thermophysical properties and values of nanofluids.

Nanofluid	Temp (K)	Viscosity (mPa.s)	Thermal Conductivity (W/mK)	Density (kg/m^3^)	Specific Heat (J/kgK)	Particle Mass Concentration (%)
Al_2_O_3_	293	1.018	0.582	1007	4149	1.16
	303	0.844	0.596	1004	4144	
	313	0.67	0.612	1001	4142	
	323	0.605	0.638	997	4141	
TiO_2_	293	0.840	0.612	1006	4147	1.16
	303	0.84	0.621	1004	4142	
	313	0.687	0.634	1001	4139	
	323	0.567	0.658	997	4139	
ZnO	293	1.023	0.584	1012	4127	1.66
	303	0.872	0.605	1009	4121	
	313	0.699	0.643	1006	4119	
	323	0.559	0.651	1002	4118	
CuO	293	1.045	0.618	1014	4165	1.92
	303	0.865	0.635	1011	4167	
	313	0.730	0.654	1008	4171	
	323	0.615	0.672	1004	4175	
ZnO + CuO	293	1.035	0.601	1017	4125	1.78
	303	0.870	0.620	1015	4127	
	313	0.725	0.648	1011	4130	
	323	0.595	0.662	1007	4133	

**Table 3:** Technical specifications of the automotive radiator used in this study [28].

Parameter	Value
Brand/Model	KALE/ABB
Honeycomb (core) dimensions (W × H × D)	228 × 196.4 × 45 mm
Channel dimensions—exterior	3 × 37.2 mm
Channel wall thickness	0.6 mm
Channel dimensions—interior (H × W)	1.8 × 36 mm
Channel hydraulic diameter	~3.4 mm
Number of channels and Fin pitch (fins per inch)	16 and 10 fpi
Fin type and material	Louver and Aluminum

**Table 4:** Cooling loads obtained from the experimental study.

TEST NO	Flow Rate Q (L/min)	Air Velocity V_air_ (m/s)	Pure Water (PW) Cooling Load Q_cool_ (kW)	ZnO + PW Cooling Load Q_cool_ (kW)	ZnO + CuO + PW Cooling Load Q_cool_ (kW)
1	17	6	9.8	12.9	12.8
2	17	8	10.8	13.9	13.2
3	17	10	10.8	14.5	14.3
4	19	6	10.9	13.1	13.6
5	19	8	11.8	14.0	14.7
6	19	10	12.8	16.0	16.0
7	21	6	11.2	13.9	16.4
8	21	8	11.7	14.2	17.4
9	21	10	12.9	18.2	19.6

**Table 5:** Cooling loads of the working fluids used in this study.

Fluid	Fluid Outlet Temperature T_cool,out_ (°C), CFD	Fluid Cooling Load, Q_cool_ (kW), CFD
Pure Water	61.3	12.3
CuO + ZnO + Pure Water	57.9	17.7
ZnO + Pure Water	57.6	19.0
Al_2_O_3_ + Pure Water	56.0	20.1
TiO_2_ + Pure Water	55.6	20.8

**Table 6:** Uncertainties of variables measured.

No	Instrument	Range and Variable Measured	Total Uncertainty	Uncertainty	
				Min	Max
**1**	Temperature Sensor	−40, +125 °C Fluid inlet temperature, T_in_	*U Fixed*, *T in* = 1.02 °C *UT in* = $\sqrt{U_{Fixed,T_{in}}^{2}+U_{Random,T_{in}}^{2}}$ = $\sqrt{1.02^{2}+0^{2}}≅$ ±1.02 °C	1.068%	1.071%
**2**	Temperature Sensor	Fluid outlet temperature, T_out_	$U_{T_{out}}$ = $\sqrt{1.02^{2}+0^{2}}≅$ ±1.017 °C	1.133%	1.369%
**3**	Manometer	0–1 bar, Pressure drop, Δ*P*	*U*_Δ_*_P_* = 1 × $\frac{0.4\%}{10\;K}$ × (90 − 20) ≅ ±0.0275 bar	12.49%	-
**4**	Flowmeter	1–90 L/min ∀˙ Volume flow rate,	$U_{\forall }^{.}$ = $\sqrt{{\left(0.1\right)}^{2}+{\left(0\right)}^{2}}≅$ ±0.1 L/min	0.040%	1.0%
**5**	Thermophysical Properties	Thermal Conductivity, *k* Dynamic Viscosity, *µ* Density, ρ Specific Heat, *C_p_*	$\frac{U_{k}}{k}$ = $\sqrt{{\left(0.05\right)}^{2}+{\left(0.0354\right)}^{2}}=$ ±6.13% $\frac{U_{µ}}{µ}$ = $\sqrt{{\left(0.05\right)}^{2}+{\left(0.0527\right)}^{2}}=$ ±7.26% $\frac{U_{\rho }}{\rho }$ = $\sqrt{{\left(0\right)}^{2}+{\left(0.0019\right)}^{2}}=$ ±0.19% $\frac{U_{C_{p}}}{C_{p}}$ = $\sqrt{{\left(0\right)}^{2}+{\left(0.025\right)}^{2}}=$ ±2.5%	0.19%	7.26%

**Table 7:** Uncertainty of results calculated.

No	Result	Maximum Uncertainty
**1**	Mass flow rate, *m*˙ = ρ∀˙	$\frac{U_{\Delta T}}{\Delta T}$ = ${\left[{\left(\frac{\partial \dot{m}}{\partial \rho }.\frac{U_{\rho }}{m}\right)}^{2}+{\left(\frac{\partial \dot{m}}{\partial \dot{\forall }}.\frac{U_{\dot{\forall }}}{\dot{m}}\right)}^{2}\right]}^{0.5}$ = [(0.03%)^2^ +(1.0%)^2^]^0.5^ = 1.00%
**2**	Temperature difference in fluid from inlet to outlet, Δ*T* = *T_out_* − *T_in_*	$\frac{U_{\Delta T}}{\Delta T}$ = ${\left[{\left(\frac{\partial \Delta T}{{\partial T}_{out}}.\frac{U_{T_{out}}}{\Delta T}\right)}^{2}+{\left(\frac{\partial \Delta T}{{\partial T}_{in}}.\frac{U_{T_{in}}}{\Delta T}\right)}^{2}\right]}^{0.5}$ = ${\left[{\left(\frac{1.02}{10}\right)}^{2}+{\left(\frac{1.02}{10}\right)}^{2}\right]}^{0.5}$ = 14.43%
**3**	Heat transfer, $\dot{Q}$ = *m*˙ *c_p_*Δ*T*	$\frac{U_{\dot{Q}}}{\dot{Q}}$ = ${\left[{\left(\frac{\partial \dot{Q}}{\dot{\partial m}}.\frac{U_{\dot{m}}}{\dot{Q}}\right)}^{2}+{\left(\frac{\partial \dot{Q}}{{\partial C}_{p}}.\frac{U_{C_{p}}}{\dot{Q}}\right)}^{2}+{\left(\frac{\partial \dot{Q}}{\partial \Delta T}.\frac{U\Delta T}{\dot{Q}}\right)}^{2}\right]}^{0.5}$ = ${\left[{\left(1.0\right)}^{2}+{\left(0.1\right)}^{2}+{\left(14.43\right)}^{2}\right]}^{0.5}$ = 3.93%

## Data Availability

The original contributions presented in this study are included in the article. Further inquiries can be directed to the corresponding author.
